# Why are we doing this alone? A collaborative framework for LDT development and validation

**DOI:** 10.1128/jcm.01867-25

**Published:** 2026-03-19

**Authors:** James E. Kirby, Ramy Arnaout

**Affiliations:** 1 Department of Pathology, Beth Israel Deaconess Medical Center 1859 , Boston, Massachusetts, USA; 2 Harvard Medical School 1811 , Boston, Massachusetts, USA; Medical College of Wisconsin, Milwaukee, Wisconsin, USA

**Keywords:** regulation, molecular diagnostics, validation, verification, LDT, clinical microbiology, analytical validation, laboratory developed test

## Abstract

Laboratory-developed tests (LDTs) play a critical role in meeting unmet diagnostic needs, particularly for rare infections and high-acuity or immunocompromised patient populations. However, current US regulatory and reimbursement frameworks have constrained innovation and delayed implementation of many essential laboratory diagnostic tests. Here, we propose a pragmatic, collaborative model focusing on infectious disease molecular diagnostics that maintains analytical rigor while allowing clinical validity to be supported by evidence from the medical literature and clinical judgment. To address the resource constraints faced by hospital laboratories, we envision a voluntary, national repository where laboratories and manufacturers contribute standardized analytical and, where appropriate, clinical validation data for both newly developed and modified Food and Drug Administration (FDA)-cleared assays. Data elements such as accuracy, limit of detection, analytical measurement range, analytical specificity, and inclusivity compared with reference determinations would be aggregated within groups representing technically identical assays, enabling cumulative validation and shared use of high-quality evidence. By leveraging these shared data sets, hospital laboratories could implement LDTs in-house with minimal redundant validation, enabling broader access to testing near the point of patient care and faster turnaround times. Manufacturers could use the same data to support applications for expanded intended use of existing FDA-cleared and -approved tests. This framework would strengthen hospital laboratory diagnostic capacity, accelerate test implementation, and improve patient outcomes and healthcare system efficiency.

## COMMENTARY

In the United States, clearance or approval by the Food and Drug Administration (FDA) applies to a specific intended use, defined by the analyte, specimen type, patient population, and clinical indication. The scope of FDA authority over *in vitro* diagnostics is defined by statute, with Congress establishing the regulatory framework and the FDA implementing it through guidance and regulation. Manufacturers must demonstrate both analytical and clinical validity, with the extent of required clinical data varying by regulatory pathway. Under the 510(k) pathway, clinical validity may be supported by demonstrating substantial equivalence to a legally marketed predicate device for the same intended use, whereas under the *De Novo* classification, clinical validity must be independently established for novel intended uses or technologies. Establishing clinical validity through prospective clinical studies can be costly and time-consuming, particularly when no appropriate predicate exists. As a result, manufacturers may pursue clearance for high-volume indications, such as cytomegalovirus (CMV) blood viral load testing in solid organ transplant recipients, but are unlikely to seek expanded clearance for the same assay in other immunocompromised populations or specimen types, despite shared pathophysiology and clear clinical relevance, because doing so would require additional clinical validity studies with limited commercial return.

Yet, there remain compelling diagnostic needs for diagnosis and management of rare or atypical infectious indications. Many of these needs are addressed through laboratory-developed tests (LDTs) offered by reference laboratories or through modification of FDA-cleared or -approved assays, for example, using an existing assay on an alternative specimen type. However, these LDTs are generally not supported by the level of clinical validity data required for FDA approval or clearance. For example, it is unlikely that any reference laboratory has formally demonstrated the clinical utility of CMV viral load testing on BAL fluid. At best, such laboratories have implemented the assay on this specimen type based on perceived need and/or literature review. Regardless, because reference laboratories typically lack access to complete clinical data and testing may be performed on a rare infectious analyte or an infrequently procured specimen type (e.g., CMV in vitreous fluid), their clinical validation cannot be other than limited and would fall far short of the current FDA threshold for market authorization. Therefore, we note in practice, and in contrast to the FDA clearance and approval framework for infectious disease molecular diagnostics, LDTs are generally validated by specimen type and analyte, with clinical interpretation, in practice, left to the ordering clinician, rather than testing being restricted to single or limited “intended-use” indications.

This approach is highly appropriate in many circumstances, and its use and interpretation can be supported by the medical literature. There is ample evidence, for example, that CMV can cause end-organ damage at a number of body sites which can be investigated through nucleic acid amplification test (NAAT)-based diagnostics. This medical knowledge provides a strong rationale for offering such a test under the LDT framework, even if the supporting data do not meet current FDA market authorization standards, and interpretation of results for some analytes is not fully resolved (e.g., CMV viral load cutoffs in BAL fluid for predicting significant lung involvement are not yet firmly established [1, 2]). Nevertheless, despite supporting a broad reference laboratory network for infrequently performed tests, this system has notable drawbacks. Sending specimens to a reference laboratory introduces delays that can result in both clinical and financial harm. In the CMV example, a rapid and accurate diagnosis can focus the diagnostic evaluation, confirm the early need for potentially life-saving antiviral therapy, or, alternatively, allow its early discontinuation, avoiding potential drug toxicities, and help redirect workup if CMV is excluded. Such a timely diagnosis shortens hospitalization, reduces costs, and helps avoid unnecessary testing or procedures.

Our guiding principle for infectious disease diagnostics should be delivery of high-quality, clinically relevant testing as rapidly as possible. Achieving this goal requires that testing be performed as near to the point of patient care as feasible. This is particularly critical for high-acuity patient populations and for infectious disease diagnostics, where delays in obtaining results can have life-threatening consequences. By contrast, reference laboratory testing introduces delay and logistical complexity related to long-distance specimen transport. During prolonged turnaround times, clinicians may initiate additional diagnostic testing while awaiting results, which can extend diagnostic evaluation and increase resource utilization.

The challenge, then, is how to enable timely access to advanced diagnostic testing within local, high-complexity laboratories. Three complementary strategies merit consideration:

1. Reducing the regulatory burden associated with FDA clearance without practically compromising test quality could encourage broader development and deployment of clinically meaningful assays, including on testing platforms already available in clinical laboratories. We recognize that the FDA has, in certain circumstances, demonstrated flexibility in its regulatory approach, including acceptance of spiked or archived specimens and other alternative validation strategies for low-incidence pathogens during pre-submission interactions. However, such accommodations are typically applied on a case-by-case basis and do not consistently lower the overall barriers to clearance for assays targeting rare conditions or specialized clinical indications. We also recognize that regulatory clearance represents only one of several barriers to manufacturer investment in such assays, alongside the ongoing costs of labeling, post-market surveillance and reporting requirements, and distribution, which may further limit commercial viability for low-volume indications.
2. Lowering barriers to local implementation of laboratory-developed tests could expand access to essential diagnostics tailored to specific patient populations. Streamlining of validation should remain consistent with CLIA principles for analytical validation (e.g., accuracy, precision, analytical sensitivity, and specificity) while recognizing that pooled, statistically robust validation data could satisfy these analytical performance requirements across institutions. Local laboratories would retain responsibility for verifying only those parameters materially affected by site-specific variables such as instrumentation, workflow, personnel competency, and specimen handling.
3. Leveraging real-world data and modeling to make informed predictions about how testing is likely to perform in specific patient groups prior to formal study (3, 4). Large, heterogeneous data sets can be used to assess whether patient-level or clinical factors meaningfully influence analytical or clinical performance, and, when such effects are absent, to support streamlined approaches rather than duplicative sub-group-specific studies. This strategy has been successfully applied in infectious disease diagnostics and could be extended to other areas of laboratory testing. This approach aligns with ongoing FDA initiatives that use real-world data to inform regulatory decision-making, allow application of findings across related patient populations, and, when appropriate, reduce the need for additional clinical studies.

A balanced approach that incorporates all three strategies would advance the overall goal of improving patient care through faster, evidence-based diagnosis while maintaining the high standards of analytical and evidence-based clinical validity that are foundational to laboratory medicine. The framework proposed here does not require amendment of the Clinical Laboratory Improvement Amendments of 1988 (CLIA) but could be implemented through updated guidance, interpretive frameworks, or voluntary collaborative mechanisms consistent with existing CLIA principles that already allow reliance on external analytical data and risk-based validation under medical director oversight.

Our focus here is on clinical microbiology molecular diagnostic testing, defined as the detection of pathogen DNA or RNA through quantitative or qualitative methods. In particular, we focus on NAATs, which have well-established analytical performance characteristics. *In silico* analyses can complement traditional validation approaches by assessing assay inclusivity and identifying potential off-target cross-reactivity. From a diagnostic standpoint, these features make molecular targets a particularly robust and transparent use case for the framework we propose.

Modeled on prior European practice under earlier EU diagnostic frameworks and recognizing that the current *In Vitro* Diagnostic Regulation (IVDR) has since expanded requirements, we propose that Congress or the FDA, through revised guidance, recalibrate requirements for market authorization to emphasize robust analytical performance while allowing clinical relevance to be supported by established evidence in the medical literature, rather than requiring new clinical trials in all cases (5). The essential question should be whether a test reliably detects a given virus, bacterium, or parasite in a specified specimen type. Reliance on large, costly clinical trials to establish diagnostic relevance should be minimized. In many cases, the existing medical literature and well-established clinical principles may suffice for approval. For example, it is unreasonable that the intended use of a CMV viral load assay be restricted solely to bone marrow transplant recipients simply because a dedicated trial has not been performed in other types of immunocompromised populations, despite well-documented parallels in disease pathophysiology and clinical management. Likewise, requiring extensive additional clinical validation for the same CMV assay in bronchoalveolar lavage fluid should be unnecessary if analytical performance has already been demonstrated, recognizing that targeted or pragmatic studies may still be appropriate to inform quantitative interpretation or clinical thresholds. The responsibility should instead shift to the end user, the treating clinician, who can interpret results within the context of clinical knowledge and peer-reviewed evidence and, for example, determine whether detection of CMV in BAL fluid has actionable clinical relevance (1, 6, 7). In this model, robust analytical validation provides the basis for clearance, while clinical practice and evidence determine how and where a test is applied.

We recognize that this approach could, in theory, permit marketing of tests lacking sufficient clinical justification, for example, by boutique reference laboratories. This concern is not hypothetical. The current LDT market already includes examples of molecular panels, such as broad assays for urinary flora, that have been introduced without robust evidence supporting clinical relevance or utility. Related concerns have also been raised in other clinical areas, including oncology, where some commercially available LDTs have been deployed with limited publicly available data supporting analytical validation or clinical utility. While these practices warrant scrutiny, a detailed discussion is beyond the scope of the present commentary, which is focused on high-acuity infectious disease molecular diagnostics, where unmet clinical needs are common, clinical scenarios may evolve quickly and require rapid diagnostic results, and clinical rationale is well established.

At the same time, even as clinical validity criteria are refined, implementation of critical inpatient testing should not be impeded. For example, HSV PCR testing of blood in patients with unexplained fulminant hepatitis addresses an obvious clinical need and is often adopted in high-acuity inpatient settings based on medical necessity and clinical urgency. Within our proposed framework, manufacturers could achieve more rapid market authorization for assays with clear analytical and clinical rationale. For instance, if peer-reviewed studies demonstrate that an HSV-1 and HSV-2 PCR assay currently approved for analysis of mucocutaneous specimens performs reliably on blood (8, 9), expanded intended use could be granted without requiring large new clinical trials. Similarly, PCR assays for *Pneumocystis jirovecii*, *Babesia*, *Anaplasma*, *Ehrlichia*, and *Plasmodium* could be authorized for use on rapid diagnostic platforms without the prohibitive requirement of large clinical trials for infections that are relatively uncommon in the United States but associated with substantial morbidity.

Turning to local LDT implementation, hospital-based clinical laboratories operate under significant resource constraints that limit their capacity to independently develop, validate, and maintain specialized molecular assays. Laboratories serving high-acuity, immunocompromised populations must respond to the need for essential testing not available on commercial platforms. However, they must often triage development based on limited resources or may be unable to implement LDTs at all, despite compelling clinical rationale. To support this local development, we propose that establishing LDT clinical validity should similarly emphasize insights based on the medical literature and sound clinical reasoning. The key challenge is to ensure robust analytical performance while enabling laboratories to bring LDTs online rapidly to meet urgent local needs.

Notably, clinical laboratories across the United States considering LDT development will often use the same instruments, reagents, and protocols to establish these assays, yet do so largely in isolation. Coordinating such efforts and sharing validation data could reduce duplication and improve the efficiency and consistency of LDT implementation. In addition, many LDTs arise from modifications of FDA-cleared assays, for example, adapting a test for a different specimen type, and these too could benefit from the same collaborative framework.

For example, consider a rapid sample-to-answer HSV PCR assay cleared for mucocutaneous “lesion” specimens. Most samples come from adults. However, neonates with disseminated HSV may shed virus from mucous membranes without visible lesions. Screening this vulnerable population is a critical unmet need. Yet, testing non-lesional swab specimens falls outside the FDA-cleared intended use of available methods, although the specimen matrix is not meaningfully different from that of lesion swabs in any way expected to affect PCR performance. Many laboratories under the existing regulatory framework have undertaken complete re-validation of analytical performance for this off-label use or simply do not offer the testing, leading to delays in diagnosis with potentially life-threatening consequences for affected neonates. We believe that extending intended use under these circumstances should be permissible based on literature review (10), commonly accepted scientific principles, and medical director oversight. This approach would parallel the conception of the Individualized Quality Control Plan (IQCP) (11), balancing risk and benefit in lieu of duplicative, underpowered validation studies (12, 13). Under such a risk-based framework, extension of intended use would require less additional validation when supported by concordant data from multiple laboratories using the same instrumentation or by a substantial body of peer-reviewed evidence, whereas more novel or unconventional applications would appropriately require more extensive validation.

We therefore propose a voluntary alternative in which validation data for LDTs, whether newly developed or modified from cleared methods, would be contributed to a central repository. Data from laboratories performing the same assay on the same specimen type would be aggregated with associated metadata in an assay-specific cumulative database. Over time, these shared data sets would expand as additional laboratories contribute, strengthening the robustness of collective validation.

A centralized resource of this kind would enable evidence-based evaluation of analytical performance across institutions and provide a foundation for broader implementation. Laboratories could adopt previously validated LDTs supported by pooled, statistically powered data, while manufacturers could leverage aggregated results to support applications for expanded intended use of marketed assays. Once cumulative accuracy data reach an adequate sample size and assay analytical performance is shown to be robust, a manufacturer could seek authorization for expanded intended use, and hospital laboratories could adopt the assay for a new specimen type following appropriate risk-benefit assessment under an IQCP framework with minimal confirmatory validation.

To achieve this goal, we envision a national, cloud-based repository designed to collect, curate, and share analytical validation data for both laboratory-developed and modified FDA-authorized diagnostic tests. The platform would function as a voluntary, collaborative repository open to contributions from accredited high-complexity clinical laboratories, diagnostic manufacturers, and academic partners. Such a repository could be hosted transparently at sites such as GitHub or Bitbucket (14), using structured web-based submission forms to facilitate data harmonization and providing decentralized access, document and version control, and oversight. Governance could be established through a consortium model involving professional societies such as the American Society of Microbiology, Association for Molecular Pathology, the College of American Pathologists, and the Association for Diagnostics and Laboratory Medicine, with input from or coordination with federal agencies including the Food and Drug Administration and the Centers for Disease Control and Prevention. An independent scientific advisory board would ensure data integrity, define minimum submission standards, and oversee data access policies.

Each entry in the repository would correspond to a specific assay configuration, defined by its analytical platform, specimen type, target pathogen or biomarker, and testing procedure. Participating laboratories would contribute standardized data sets encompassing key validation elements such as (i) limit of detection and analytical measurement range; (ii) accuracy assessed using appropriate comparator or orthogonal methods, when applicable; (iii) inclusivity, including *in silico* primer and probe coverage; (iv) analytical specificity; (v) precision; and (vi) specimen stability and matrix equivalency data when relevant. Data submission would be subject to repository governance, curation, and quality review to ensure methodological consistency and prevent aggregation of analytically incompatible data sets. All submissions would be de-identified and entered in a harmonized format to facilitate data aggregation and meta-analysis. Aggregated data could then be summarized in performance reports supporting expanded intended use or local LDT implementation without redundant validation.

While a national, shared repository of analytically validated laboratory diagnostic performance data does not currently exist, similar uses of real-world data have increasingly informed regulatory decision-making in other areas. In particular, the FDA has issued recent guidance clarifying that real-world evidence (RWE), including analyses derived from large, de-identified data sets, may be acceptable in drug and medical device submissions without requiring submission of individual-level patient data in all cases (15). This follows on RWE guidance to industry in 2023, recognizing real-world data and real-world evidence as acceptable components of regulatory decision-making for expanded or modified intended uses of approved medical products when the data are reliable and methodologically robust (16). Although FDA experience with real-world evidence has to date focused largely on drugs and non-diagnostic devices rather than laboratory diagnostics, appropriately curated and aggregated real-world data could likewise inform assessment of diagnostic test performance (3). We therefore propose applying these principles in the infectious disease molecular diagnostics space, leveraging shared analytical and real-world clinical validation data to support risk-based implementation and inform regulatory decision-making.

The sustainability of such a repository could be ensured through a hybrid funding model combining several complementary mechanisms, noting that total costs are likely to be relatively low. Participating laboratories and manufacturers might contribute through subscription-based access scaled to institutional size and usage volume. Public-private partnerships could be encouraged, with diagnostic companies providing funding or data in exchange for access to aggregated analytical insights relevant to their platforms. Additional support could be derived from competitive grants from agencies such as the National Institutes of Health, the Biomedical Advanced Research and Development Authority, or the CDC, and from foundations committed to advancing clinical diagnostics. Together, these complementary mechanisms would provide diversified and durable support for long-term repository maintenance.

Participation in such a repository would need to offer clear value to contributing laboratories. Incentives could include reciprocal access to pooled validation data, reduced local validation burden through abbreviated verification supported by cumulative validation evidence, and professional recognition of contributing laboratories through citable data sets and consortium reporting. Importantly, lowering barriers to participation through streamlined submission workflows would be essential to ensure feasibility within resource-constrained clinical laboratories. Adoption could be further encouraged by alignment with emerging journal and professional society expectations for data transparency, analogous to established norms for genomic sequence, structural biology, and high-throughput screening data deposition. For example, deposition of analytical validation data sets in the repository could be encouraged, or eventually required, for publication of validation-focused manuscripts. Such deposition would also align with NIH Data Management and Sharing Policy requirements (17), providing investigators with a practical mechanism to satisfy grant-mandated primary data sharing obligations.

As a practical next step, this framework could be piloted through a limited, voluntary consortium of high-complexity clinical laboratories performing analytically identical assays on shared platforms. Initial leadership could reasonably arise from a small group of academic clinical laboratories with established expertise in LDT development and validation, operating through a steering group to pilot scope, data standards, and participation criteria. This early, laboratory-led phase would be intentionally limited in scale, with broader professional society endorsement and formal governance considered only after feasibility and value are demonstrated through pilot efforts. Participating laboratories would contribute analytical validation data using predefined, structured formats to facilitate cross-site comparison and performance evaluation. Initial efforts could focus on a limited number of well-defined, high-acuity molecular assays where analytical performance characteristics are well understood, and clinical rationale is strong. Early engagement with regulatory stakeholders, including the FDA, could occur in parallel to ensure alignment with regulatory science initiatives and priorities and to inform future guidance. Lessons learned from such pilot efforts could then inform broader professional society endorsement and phased framework expansion.

Importantly, engagement with regulatory science initiatives could further strengthen this framework. The FDA has participated in collaborative community models through public-private partnerships such as the Medical Device Innovation Consortium’s Pathology Innovation Collaborative Community, which is focused on advancing regulatory science in artificial intelligence and digital pathology. Similar initiatives could provide a forum for laboratories, manufacturers, professional societies, and regulators to evaluate emerging approaches and data standards. Participation in such forums could help define best practices for data aggregation, assess sources of inter-laboratory variability, and inform future guidance.

The time for reform is now. The current regulatory framework for diagnostic testing, conceived in an earlier era, no longer aligns with the pace of molecular innovation, the increasingly nuanced understanding of disease, and the realities of modern patient care. In the wake of the recent *ACLA v. FDA* decision vacating expanded FDA oversight of LDTs (18), the field has an opportunity to recalibrate oversight in a manner grounded in analytical best practices and responsive to real-world clinical needs. Our proposed framework offers a balanced approach that maintains analytical rigor while promoting timely diagnostic access. We call on federal agencies, including the FDA and CMS, together with professional societies, to establish a modernized system that prioritizes analytical validity as the foundation for test clearance, leverages existing medical evidence for clinical justification, and supports robust LDT implementation at the local hospital level. A central component of this effort would be the creation of a collaborative validation data repository that harnesses the collective expertise of the laboratory medicine community and its diagnostic partners. Together, these measures would accelerate innovation, reduce redundancy, and ensure that critically ill patients receive timely access to high-quality infectious disease diagnostics.
